# Environmental DNA Sequencing Reveals a Highly Complex Eukaryote Community in Sansha Yongle Blue Hole, Xisha, South China Sea

**DOI:** 10.3390/microorganisms7120624

**Published:** 2019-11-28

**Authors:** Yueteng Liu, Hui He, Liang Fu, Qian Liu, Zuosheng Yang, Yu Zhen

**Affiliations:** 1 Key Laboratory of Marine Environment and Ecology, Ministry of Education, Qingdao 266100, China; lyt0458@163.com; 2 College of Environmental Science and Engineering, Ocean University of China, Qingdao 266100, China; 3 College of Marine Life Science, Ocean University of China, Qingdao 266003, China; hehui@ouc.edu.cn (H.H.); raulliuqian@163.com (Q.L.); 4 Sansha Trackline Institute of Coral Reef Environment Protection, Sansha 573199, China; trackchina@163.com; 5 College of Marine Geosciences, Ocean University of China, Qingdao 266100, China; zshyang@ouc.edu.cn; 6 Laboratory for Marine Ecology and Environmental Science, Qingdao National Laboratory for Marine Science and Technology, Qingdao 266237, China

**Keywords:** marine blue hole, Entoprocta, eukaryote, high-throughput sequencing, SSU rDNA

## Abstract

We report an Illumina high-throughput sequencing protocol of eukaryotic microbes in the world’s deepest marine blue hole, Sansha Yongle Blue Hole, Xisha, South China Sea. The variable V9 region of small subunit (SSU) rDNA, was sequenced using this approach from the waters of blue hole and outer reef slope. 917,771 unique eukaryotic 18S rRNA gene sequences and 6093 operational taxonomic units (OTUs) were identified. Significant differences in the eukaryotic composition were observed between the blue hole and outer reef slope, and the richness in the blue hole was much higher than that in the outer reef slope. The richness and diversity of eukaryotes in the blue hole were both lowest at 60 m and highest at 100 m depth. Eukaryotic microalgae assemblages dominated by Dinophyceae were the most abundant in the 10–20 m water column in the hole. Fauna was the main group at and below a depth of 60 m, where Araneae and Cyclopoida were dominant in the 60 m and 80 m water layer, respectively. There was a large number of Entoprocta at a depth of 180 m in the hole, where little oxygen was detected. Turbidity and nitrite concentration had a significant effect on the eukaryote community structure (*p* < 0.01).

## 1. Introduction

As special geological units formed in the carbonate bedrock, marine blue holes are located below the sea level and are dark blue in color. At present, marine blue holes have been found in the oceans and seas all over the world, such as the Saipan Blue Hole in the Pacific Ocean, Dahab Blue Hole in Egypt and the Dean’s Blue Hole in the Bahamas. Most marine blue holes in the world belong to the offshore blue hole which are wholly submerged beneath the seafloor. Some of blue holes can exchange water with the open sea by tides [1], while the exchange between some blue holes and the open seas is limited, resulting in the relatively stable internal environment and unique physical–chemical characteristics, such as a strong thermo-halocline, a highly stratified water column, and thick anoxic and hydrogen sulfide-rich layers [2,3,4,5,6,7,8].

The relatively independent geographical location and special physical–chemical characteristics of marine blue holes create a unique biological community structure [3,9]. Since the 1980s, Iliffe and Kornicker have investigated a series of blue holes in Bermuda, Bahamas and the coast of Mexico, and found that Crustaceans are obviously dominant and have high biodiversity in these holes, and many species are unique in the holes and not found in the surrounding waters [1]. Since then, eukaryotes in the blue holes have been widely investigated. The Sansha Yongle Blue Hole in the South China Sea is the deepest (300.89 m) known blue hole in the world. Studies have found that it has no major connection with the adjacent ocean and that the water column becomes a dark, anoxic, hydrogen sulfide-rich environment below 100 m [10]. The biological community structure and diversity in this unique environment has drawn the attention of many scientists. For example, Chen’s group identified 41 mesoplanktonic species and 14 groups of planktonic larvae in the hole, with the dominant species being *Oithona attenuata*, followed by *Oithona rigida* and *Scolecithricella longispinosa*, during both daytime and nighttime [11]. However, the composition of eukaryotic microbes in this unique environment has not yet been reported.

To date, eukaryotic algae and fauna in marine blue holes have been widely studied by peer scholars; however, due to method limitations, most studies have only identified and described the species found in anchialine caves [12,13]. The Yongle Blue Hole is far from the mainland and located on the continental slope in a deep-water area of the South China Sea, and many shallow reefs and submerged reefs around it make access and sampling very difficult. Therefore, it is not easy to conduct the bulk sampling necessary for traditional methods. The development of high-throughput sequencing technology has provided a new approach to studying the eukaryote community [14,15,16]. Compared with traditional methods, high-throughput sequencing technology requires less volume and can obtain a comprehensive analysis of biological community characteristics [14,17,18]. This paper is the first to analyze the eukaryote community characteristics in the water column of the Sansha Yongle Blue Hole in the South China Sea, using 18S rRNA gene-based Illumina high-throughput sequencing, to reveal the structure and diversity of the eukaryote community in the hole and provide a basis for community and function analyses of marine blue holes.

## 2. Materials and Methods

### 2.1. Sample Collection

The working pontoon was built on the surface of the Sansha Yongle Blue Hole in March 2017, and a winch was installed on the pontoon. Then, 5 L of water was collected by Niskin water samplers at depths of 0 m, 10 m, 20 m, 60 m, 80 m, 90 m, 100 m, 150 m and 180 m in the blue hole. In addition, 3 L of water was collected from a boat at depths of 0 m and 50 m in the outer reef slope adjacent to the blue hole (Table 1). After collection, all samples were filtered through 75 μm mesh nets to remove larger organisms and particles, and then filtered through 0.22 μm filters to collect the eukaryotes. The membranes were placed into sterile frozen pipes and immediately frozen in liquid nitrogen for further nucleic acid extraction.

### 2.2. Determination of Environmental Parameters

Environmental parameters (pH, salinity, temperature, dissolved oxygen concentration and turbidity) of each layer were recorded with an Alec ASTD102 self-contained CTD (ALEC Electronics Co. LTD, Japan) and an AAQ171 Real-time profiler (JFE Advantech, Japan) in situ. The silicate, phosphate, nitrate, nitrite, and ammonium concentrations were cited from Yao′s group [19].

### 2.3. Nucleic Acid Extraction

The membranes stored in liquid nitrogen were cut into small pieces and placed into 2 mL centrifuge tubes. Total genomic DNA was extracted using the cetyltrimethyl ammonium bromide (CTAB) method [20]. The integrity, concentration, and purity of the DNA were determined by Picodrop microliter UV/Vis spectrophotometer (Picodrop, Cambridge, UK) and agarose gel electrophoresis analysis. The extracted DNA was stored at −20 °C for high-throughput sequencing.

### 2.4. High-Throughput Sequencing

The V9 region of the 18S rRNA gene was amplified using universal primers (1380F: 5′-CCCTGCCHTTTGTACACAC; 1510R: 5′-CCTTCYGCAGGTTCACCTAC) [21]. We used 200 ng DNA of each sample for the polymerase chain reaction (PCR). The amplicons were verified by 2% agarose gel electrophoresis and then mixed into equal amounts based on product concentrations. The Gel Extraction Kit (QIAGEN, Germany) was used to purify the amplicons, then the amplicons were pooled to construct the libraries using the TruSeq^®^ DNA PCR-free Sample Preparation Kit. The barcode was ligated to the 5′ ends of primers to distinguish each sample. After Qubit and qPCR quantitative detection, paired-end sequencing of the amplicons was performed on a HiSeq2500 PE250 sequencer platform (Novogene, Beijing, China).

### 2.5. Bioinformatic Analysis

After removing the barcodes and primers, the raw data were merged using the fast length adjustment of short reads (FLASH) method [22]. Raw reads were cut from the first base position of three consecutive low-quality bases (phred quality score < 20). Only the front parts were leaved and then filtered out reads of which the continuous high-quality base length was less than 75% of the length of reads using Quantitative Insights into Microbial Ecology (QIIME) [23]. After quality control, clean reads were obtained for further bioinformatics analysis. Uparse (v7.0.1001, http://drive5.com/uparse/) was used to cluster the clean reads into operational taxonomic units (OTUs) with a 97% similarity cutoff [24]. Chimeras have already been identified and removed from the dataset using Uparse cluster_otus command. The most common sequence in each OTU was chosen as the representative sequence and assigned with the Silva database (http://www.arb-silva.de/, Version 128) [25] to obtain the annotation information of each OTU. All raw data on the eukaryote 18S rRNA gene generated in this study have been submitted to the NCBI Sequence Read Archive under the accession number PRJNA548451.

QIIME (Version 1.7.0) was used to calculate the Chao1 index and Shannon index and to determine the rarefaction curve of each sample. The unweighted pair group method with arithmetic mean (UPGMA) clustering was also conducted by QIIME. A Venn diagram was drawn using the R software (Version 3.5.1) VennDiagram package. Canonical correspondence analysis (CCA) between the eukaryote community and environmental parameters was determined by the R software (Version 3.5.1) vegan package, and verified by the Monte Carlo permutation test.

## 3. Results

### 3.1. Environmental Parameters in Sampling Stations

**Figure 1:**
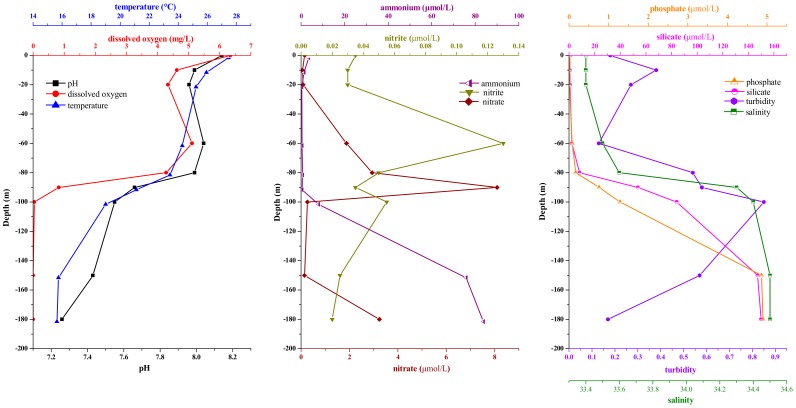
Environmental parameter profiles in the Sansha Yongle Blue Hole in March 2017.

The environmental parameters at the nine sampling stations in the Sansha Yongle Blue Hole are shown in Figure 1. Temperature, pH and dissolved oxygen concentration clearly decreased with increasing depth, while salinity, silicate concentration, phosphate concentration, and ammonium concentration increased significantly with increasing depth. Turbidity, nitrate concentration and nitrite concentration peaked at depths of 100 m, 90 m, and 60 m, respectively. The dissolved oxygen concentration decreased to less than 1 mg L^−1^ below 90 m, indicating that the Yongle Blue Hole began to become anoxic at this depth.

### 3.2. Diversity and Composition Analysis of Eukaryote Community

**Figure 2:**
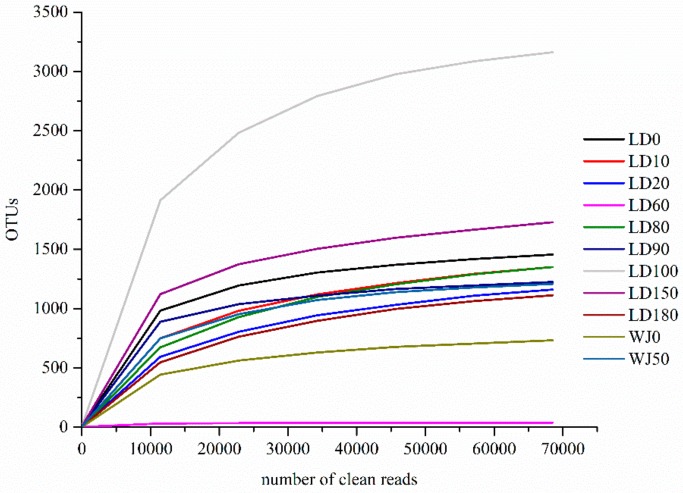
Rarefaction curve of each sample.

A total of 917,771 clean reads were obtained from 11 samples, and the number of clean reads in each sample was between 68,851 and 93,308. According to the 97% similarity cutoff, 6093 OTUs were obtained, and the number of OTUs in each sample was in the range of 41–3302. The coverages were greater than 99.0%, and the rarefaction curves gradually became flat, indicating that the sequences retrieved from this study could reflect the eukaryote community characteristics in this area (Figure 2).

Chao1 showed that the richness of eukaryotes in the Sansha Yongle Blue Hole was generally greater than that in the outer reef slope (Table 2). The diversity of the eukaryote community in the surface layer of the hole was significantly greater than that in the surface layer of the outer reef slope. From depths of 0 m to 50 m, the diversity of the eukaryote community in the hole decreased significantly with increasing depth, in contrast with the diversity of the eukaryote community in the outer reef slope. The richness and diversity of eukaryotes in the hole were lowest at a depth of 60 m and highest at a depth of 100 m.

**Figure 3:**
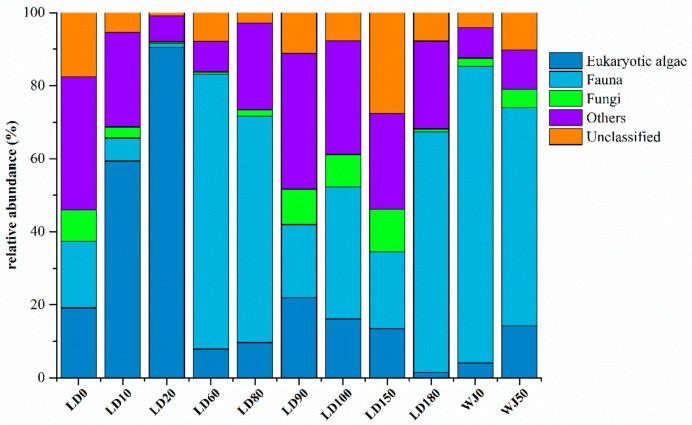
Relative abundance of eukaryotes in different samples.

The eukaryotes in the 11 samples from the hole and the outer reef slope were grouped into 71 phyla, 177 classes, 276 orders, 294 families, 541 genera and 573 species. The proportions of eukaryotic algae and fauna in each sample were 1.51–90.49% and 1.06–81.27%, respectively (Figure 3). In general, the relative abundance of eukaryotic algae increased significantly with depth from 0 m to 20 m in the hole, and reached its maximum value at a depth of 20 m (90.49%). Fauna was the main group (20.02–75.30%) in the surface layer and from 60 m to 180 m in the hole, whereas it occupied the dominant position (59.77–81.27%) from 0 m to 50 m in the outer reef slope. The relative abundance of fungi in each sample ranged from 0.49% to 11.74%, representing only a very small proportion of the total eukaryotes.

**Figure 4:**
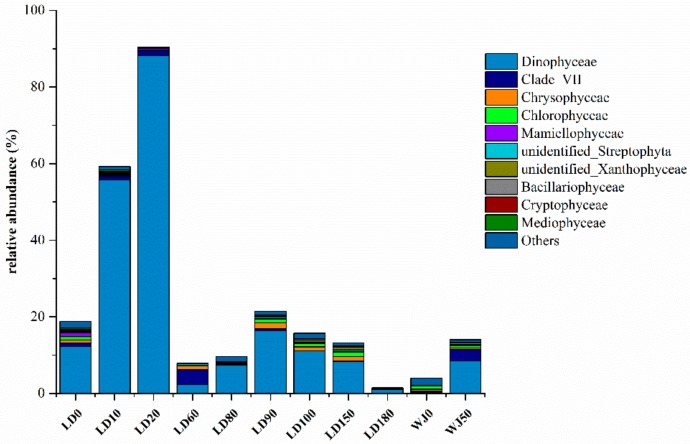
Relative abundance of eukaryotic algae at the class level.

The relative abundance of the eukaryotic algae community at the class level is shown in Figure 4. A total of 22 phyla and 57 classes of eukaryotic algae were identified in the 11 samples. Dinophyceae which peaked at depth of 20 m (88.23%) was the main group of eukaryotic algae in the hole. The distribution characteristic of Dinophyceae was consistent with that of the eukaryotic algae community, which also verified the dominant position of Dinophyceae in the hole. Chlorophyceae and Dinophyceae were the main groups of eukaryotic algae at depths of 0 m and 50 m, respectively in the outer reef slope.

**Figure 5:**
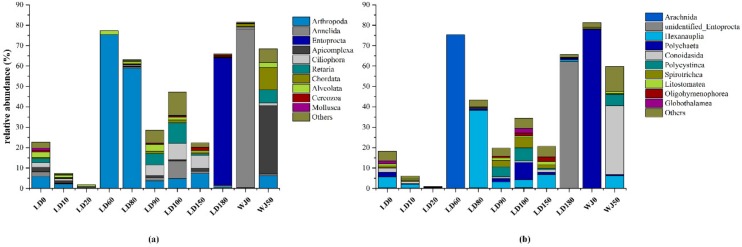
Relative abundance of fauna at different levels: (**a**) at the phylum level; (**b**) at the class level.

The relative abundance of the fauna community at the phylum and class levels are shown in Figure 5. A total of 27 phyla and 79 classes were identified in this study. Annelida, Arthropoda, Entoprocta, Apicomplexa and Ciliophora were the predominant phyla in the hole and the outer reef slope. Arthropoda was the main fauna group (58.86–75.30%) from 60 m to 80 m in the hole which featured aerobic water conditions. At the class level, Arachnida and Hexanauplia were dominant at depths of 60 m and 80 m, respectively, in the hole. Polychaeta, Hexanauplia, Polycystinea, Spirotrichea, and Entoprocta were the dominant groups from 90 m to 180 m, which featured anoxic water conditions. Notably, the relative abundance of Entoprocta was 62.35% at depth of 180 m in the hole, while its relative abundance at other depths in the hole was only 0–0.26%. Entoprocta was also not detected in the outer reef slope. The dominant groups at depths of 0 m and 50 m in the outer reef slope were observed to be quite different: Polychaeta was dominant at a depth of 0 m with a relative abundance of 77.84%, whereas Conoidasida was dominant (33.67%) at a depth of 50 m.

**Figure 6:**
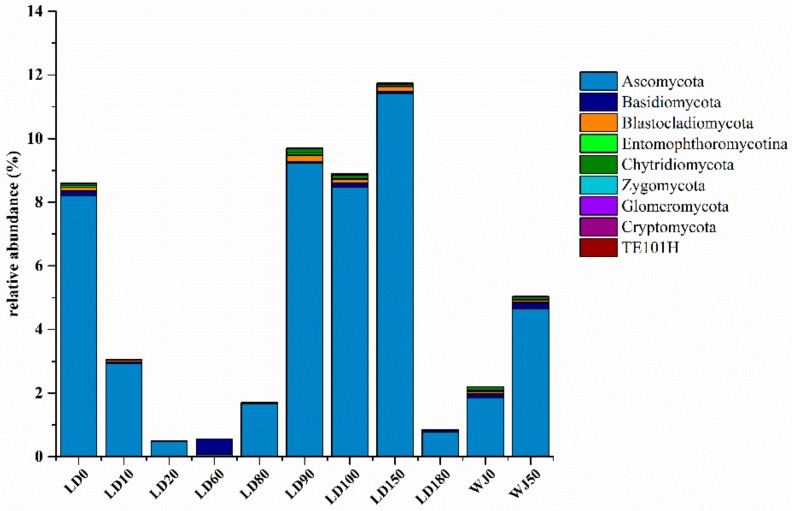
Relative abundance of fungi at the phylum level.

At the phylum level, Ascomycota was the dominant group (0.80–11.42%) of the fungal community in this study (Figure 6). In general, the relative abundance of Ascomycota in the hole was greater than that in the outer reef slope, especially in the water layers from 90 m to 150 m in the hole where its relative abundance reached 8.47–11.42%.

Among the top ten OTUs within all samples, three OTUs can be classified as eukaryotic algae, six OTUs can be classified as fauna, and only one OTU can be classified as fungi (Table 3). The three eukaryotic algae were all assigned to Gymnodiniphycidae, which belong to Dinophyceae; the abovementioned fauna belonged to Phascolosomatiformes, Araneae, Cyclopoida, Gregarinasina, Entoprocta and unclassified Arthropoda; and the fungi were assigned to Thelebolales, which belongs to Leotiomycetes. Among the top ten OTUs, only OTU1 and OTU8 were the dominant groups in the outer reef slope, and the other OTUs were the dominant groups in the hole, further highlighting the significant differences in the eukaryote community compositions between the hole and the outer reef slope.

### 3.3. Comparison of Eukaryote Community Structure

#### 3.3.1. Comparison of Eukaryote Community Structure at Different Depths in the Hole

**Figure 7:**
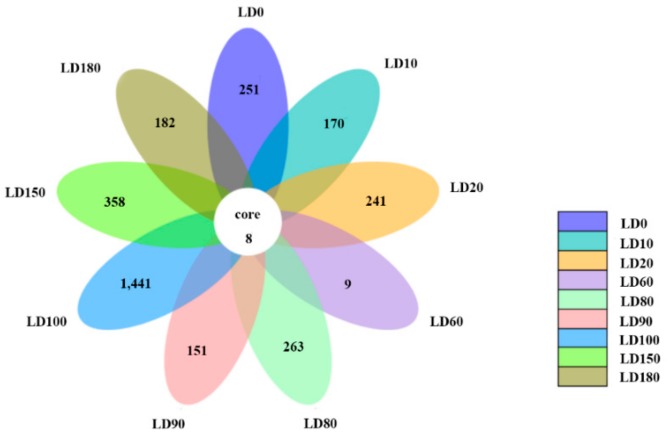
Comparison of the eukaryote communities at different water layers in the Sansha Yongle Blue Hole.

Eukaryote communities at different depths in the hole were compared (Figure 7), and only 8 species, including Dinophyta, Chlorophyta, Protalveolata, and other unclassified eukaryotes, were observed at all depths. The results also showed that the number of endemic OTUs in different layers varied from 9 to 1441, and peaked at a depth of 100 m. It is possible that a strong thermo-halocline exists between 80 m and 90 m and hinders the vertical movement of eukaryotes [11]. Furthermore the interface of aerobic and anaerobic conditions also makes the depth of 100 m a unique environmental barrier for eukaryotes.

#### 3.3.2. Comparison of Eukaryote Community Structures in the Hole and the Outer Reef Slope

**Figure 8:**
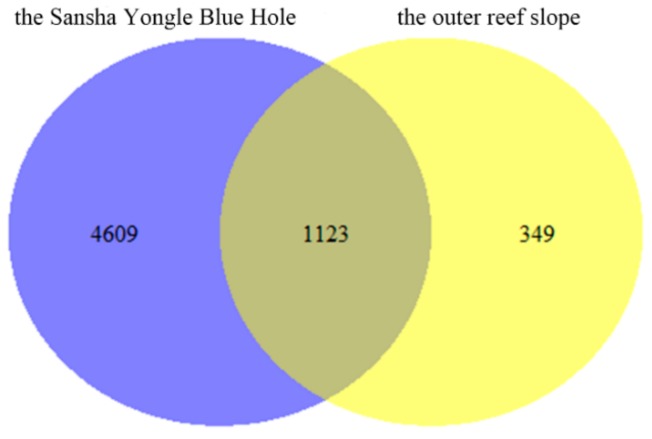
Comparison of the eukaryote communities in the Sansha Yongle Blue Hole and the outer reef slope.

The eukaryote communities in the hole and the outer reef slope were also compared in this study (Figure 8). This study identified a total of 1123 mutual species, 4609 endemic species in the hole and 349 endemic species in the outer reef slope, with the endemic species accounting for 75.80% and 5.74% of the total species in the hole and the outer reef slope, respectively. The eukaryote communities in the hole and the outer reef slope were hypothesized to be quite different, and the large number of endemic species in the hole need to be further studied.

**Figure 9:**
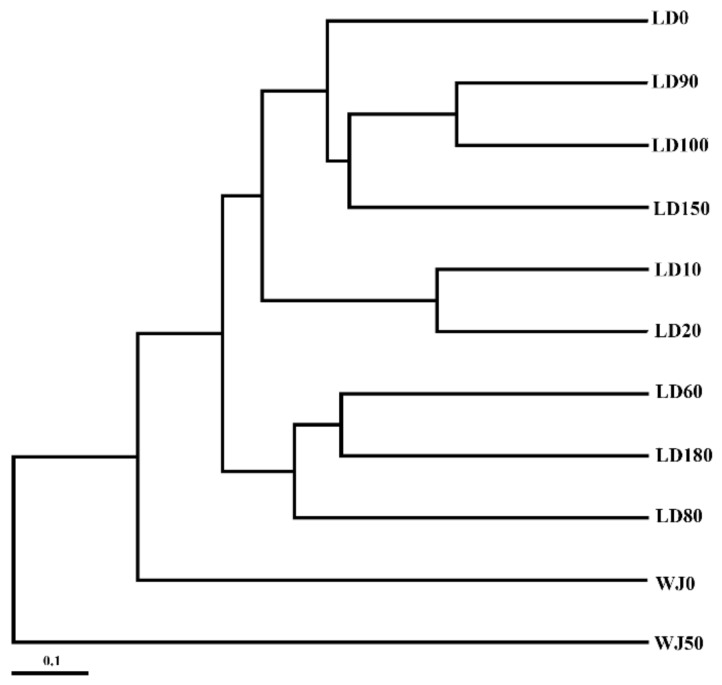
Unweighted pair group method with arithmetic mean (UPGMA) clustering of the eukaryote communities in the Sansha Yongle Blue Hole and the outer reef slope.

The comparison of eukaryote communities between the hole and the outer reef slope was also shown by UPGMA clustering. All samples can be clearly divided into two branches (Figure 9). In general, the eukaryote community in the surface layer of the outer reef slope was quite similar to that in the hole, probably due to the frequent water exchange in the surface layer.

### 3.4. Correlations between the Eukaryote Community Structure and Environmental Parameters

**Figure 10:**
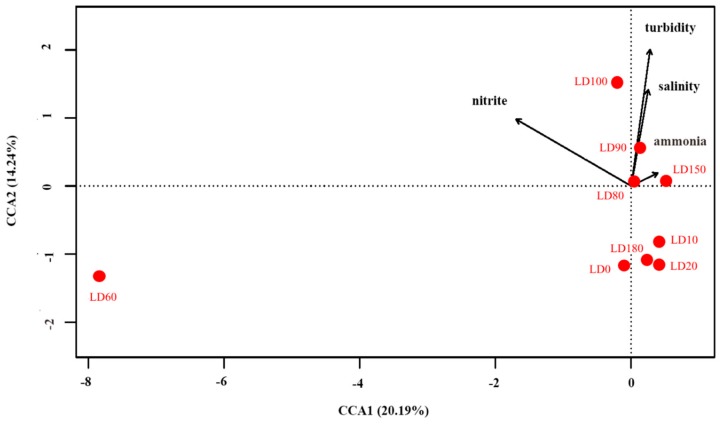
Canonical correspondence analysis between environmental parameters and eukaryote community structure.

Correlations between eukaryote community structure and environmental parameters were analyzed by R software (Version 3.5.1). Decision curve analysis (DCA) showed that canonical correspondence analysis (CCA) was suitable for this study (Figure 10). The BIOENV analysis showed that turbidity, salinity, nitrite concentration, and ammonium concentration had greater influences on the eukaryote communities than other environmental factors. The first two CCA dimensions explained 34.43% of the cumulative variances of the genotype-environmental relationship. The first axis had a positive correlation with ammonium concentration and a negative correlation with nitrite concentration. The second axis had positive correlations with turbidity and salinity. The Monte Carlo permutation test showed that turbidity and nitrite concentration significantly affected the eukaryote community in the blue hole (*p* < 0.01). Moreover, there was a significant difference between the eukaryote community structure at a depth of 60 m and at other water depths, and the results showed that nitrite concentration had a greater impact in LD60 than in other samples.

## 4. Discussion

In the present study, eukaryote community characteristics were measured in the water column of the Sansha Yongle Blue Hole, using Illumina high-throughput sequencing technology. The results showed that the relative abundance of eukaryotic microalgae dominated by Dinophyceae was greater in the water columns from 10 m to 20 m in the hole. Light intensity can affect the growth of eukaryotic algae. The growth of eukaryotic microalgae can be inhibited by excessive light, and eukaryotic microalgae usually reached its maximum biomass in subsurface layers [26]. Previous studies have documented that diatoms and Dinophyta were dominant in the South China Sea, but that the cell abundance of diatoms was much greater than that of Dinophyta [27]. However, the nutrient concentrations in the water column from 0 m to 20 m in the hole were significantly lower than those in other areas of the South China Sea [28,29,30]; therefore, Dinophyta, which is more tolerant of low nutrient concentrations than diatom, was better able to thrive in this zone [31].

Unlike the communities in the water column from 10 m to 20 m, the communities in the water column below a 60-m depth in the hole were dominated by fauna, and it was speculated that a strong thermo-halocline at depth of 50 m [10] prevented the fauna from migrating from the deeper water layers to the shallower layers. A total of 26 phyla and 78 classes of fauna were identified in the hole, and Araneae and Cyclopoida which belong to Arthropoda were the main groups in the aerobic layers from 60 m to 80 m. Using the traditional microscopic method, Chen′s group found that the dominant fauna were *Oithona attenuata*, *Scolecithricella longispinosa* and *O. rigida* in the hole, consistent with our results [11]; moreover, our results also indicated that high-throughput sequencing technology could be used to obtain a more comprehensive understanding of the eukaryote community characteristics in the hole. Lejzerowicz′s group used both traditional morphological and high-throughput sequencing technology to compare the metazoan community characteristics of a fish farm in Scotland [32], and the results showed that the data obtained based on the high-throughput sequencing method not only included the macrofaunal species that dominated in the morphological samples but also included the small-sized species (<1 mm), thereby enriching the morphological analysis results. Polychaeta, Hexanauplia, Polycystinea, Spirotrichea, and Entoprocta were the dominant groups in the anaerobic water column below 90 m, and this community composition was quite different from the dominant groups in the aerobic water layers, possibly due to the dissolved oxygen concentration. Analysis of the eukaryote community structures in the oxic environments, interface between the oxic and anoxic environments, and anoxic environments in the Cariaco Basin in Venezuela, the world’s largest anoxic marine basin also indicated that the dissolved oxygen concentration had a significant impact on the distribution of eukaryotes [33].

**Figure 11:**
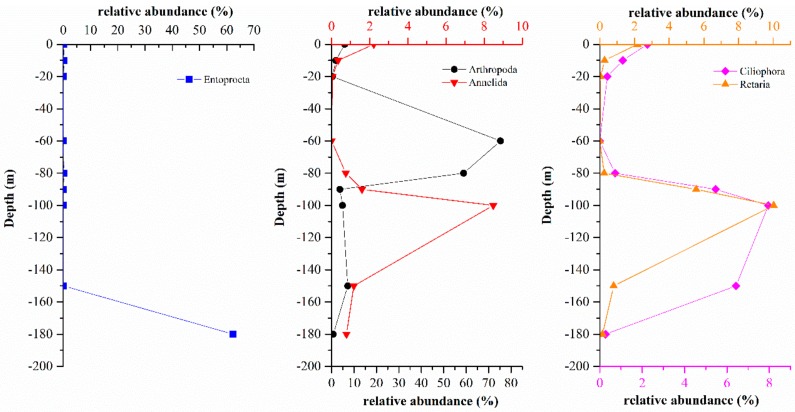
Relative abundances of Entoprocta, Arthropoda, Annelida, Retaria, and Ciliophora in the Sansha Yongle Blue Hole at different depths.

Compared with the eukaryote compositions in the water layers above 150 m, the eukaryote compositions at a depth of 180 m in the hole were significantly different. Entoprocta was absolutely dominant (62.35%) at a depth of 180 m and all the identified Entoprocta belonged to Loxosomatidae, 99.62% of which belonged to *Loxosomella-plakorticola*. However, the relative abundance of Entoprocta was only 0–0.26% in the water columns above 150 m in the hole. With increasing depth, the abundances of Arthropoda, Annelida, Retaria, and Ciliophora which were relatively high in the hole, first decreased, then increased and finally decreased (Figure 11), and this pattern was significantly different from the distribution of Entoprocta. Therefore, it was speculated that a large number of Entoprocta might be attached to the wall at a depth of 180 m and that their budding or residual bodies were released at this depth, making the abundance of Entoprocta relatively high. To date, Entoprocta has been found to include approximately 180 species, and most live in marine environments and often live symbiotically with marine invertebrates [34,35]. Entoprocta is widely distributed around the world, from tropical to temperate to polar seas and from intertidal to deep-sea waters [36], but thorough studies have been performed only on the Entoprocta in the shallow waters of eastern Europe, the United States, and Japanese islands [34,37,38]. Until now, Entoprocta has not been reported in the South China Sea. The first report of *Loxosomella-plakorticola*, a new Loxosomella species that existed in symbiosis with sponges, was in the water column from 10 m to 15 m in the Ryukyu Archipelago [34]. In contrast to the above-mentioned research, the *Loxosomella-plakorticola* in the Sansha Yongle Blue Hole at a depth of 180 m under absolutely anoxic conditions, yet no research has suggested that *Loxosomella plakorticola* was able to survive under anoxic conditions to date. This study could provide new ideas for exploring the adaptability of *Loxosomella-plakorticola* in such environments.

Compared with the outer reef slope, the hole exhibited a higher species richness, and significant differences in the eukaryote communities were observed between the hole and the outer reef slope, consistent with Chen’s results [11]. Studies have shown that Alveolata was the dominant group in the eukaryote communities in other areas of the South China Sea [39,40,41], which was quite different from our results. This difference highlights the uniqueness and scientific value of the marine blue hole ecosystem. The fauna in the marine blue hole encompassed a diverse range of taxa, with annelids, arachnids, chaetognaths, echinoderms, gastropods, poriferans, turbellarians, and crustaceans occupying dominant positions [8,42], as confirmed by our results.

The fine particle components (145–500 μm) of rock debris and minerals in the hole were mainly derived from debris from the nearby coral reef, and these materials were the main factors affecting the turbidity of the water [10,43]. In the present study, turbidity also had a significant effect on the eukaryote community structure in the Sansha Yongle Blue Hole. Turbidity directly affected the photosynthesis of eukaryotic algae, which indirectly affected the predation, competition, and symbiosis of other eukaryotes such as fauna and fungi [44,45]. Hydrodynamic turbulence and turbidity can regulate the community structure of fauna by influencing predators of larger invertebrates and fish [46,47]. Jack demonstrated that suspended particles can affect the competition of animals by inhibiting the growth of animals such as copepods [48]. In addition, the correlation analysis between eukaryote community structure and environmental factors showed that nitrite concentration also had a significant impact on the eukaryote community structure in the hole, similar to the findings of studies in a deep artificial lake and the Mogi–Guaçu basin [49,50].

**Figure 12:**
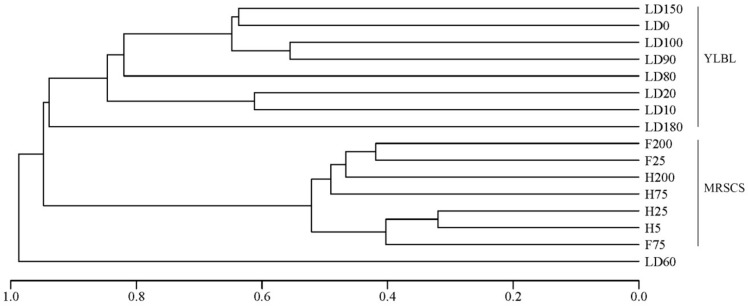
Clustering analysis of the eukaryotic community in the water column from the Sansha Yongle Blue Hole (YLBL) and the mid-region in the South China Sea (MRSCS).

In this study, we also discussed differences of eukaryote communities in the water column between the Yongle Blue Hole and other areas in the South China Sea. The 18S rRNA gene obtained in this study and another study (the water samples from the mid-region in the South China Sea) [51] were trimmed as reported above. The high-quality sequences were clustered into 10,643 OTUs at a 97% similarity cutoff. The richness and diversity of the eukaryotic community in the water column from the mid-region in the South China Sea (MRSCS) above 200 m were always greater than that in the water column from the Sansha Yongle Blue Hole (YLBL) (Table 4). Meanwhile, the richness and diversity of the eukaryotic community in all water layers were also compared, and greater richness and diversity eukaryotic communities were found in the water column from the MRSCS. Based on the clustering analysis, we found that the eukaryotic community structure in the YLBL was quite different from that in the MRSCS (Figure 12), indicating that the environmental conditions contribute significantly to the eukaryotic communities. The unique hydrological, geological and chemical characteristics of the Sansha Yongle Blue Hole, such as high sulfide concentrations, anoxic layers, restricted vertical mixing and no large scale connection with the adjacent oceans [10,19], markedly differed from the mid-region in the South China Sea, probably leading to the differences between the eukaryotic communities in these two areas.

It is debated that the DNA based sequencing methods reveal eukaryotic community structure directly, because the 18S rRNA gene sequencing output is highly affected by the huge variation in the rRNA gene copy numbers among eukaryotic species; however, Lindeque et al. have shown that the number of reads determined by a metagenetic analysis of the 18S rRNA gene using the 454 pyrosequencing platform is better correlated to eukaryote biomass than number [52]. The 18S rRNA gene high-throughput sequencing data can represent the biomass of different groups, but the number of reads for specific organisms may deviate from true values and cannot be used to estimate their absolute abundance. Further studies may be focused on the 18S rRNA gene abundance and expression of different eukaryotic groups.

## 5. Conclusions

In the present study, Dinophyceae was dominant in the water column from 10 m to 20 m in the Sansha Yongle Blue Hole, and Araneae and Cyclopoida were dominant in the water layers at depths of 60 m and 80 m, respectively, in the hole. A large number of Entoprocta were found at a depth of 180 m in the hole, which might provide new ideas for exploring the adaptability of *Loxosomella-plakorticola* to the environment. Turbidity and nitrite concentration play key roles in eukaryote community structures. Significant differences in the eukaryote community composition were observed between the hole and the outer reef slope, and the number of species in the hole was much greater than that in the outer reef slope. A large number of endemic species were found in the Sansha Yongle Blue Hole and need to be further studied. The comparison of eukaryotic communities in the water column from the Sansha Yongle Blue Hole and the mid-region in the South China Sea suggests that environmental conditions contribute significantly to the eukaryotic communities.

## Figures and Tables

**Table 1:** Sampling information.

Location	Sample Name	Depth (m)
Sansha Yongle Blue Hole	LD0	0
	LD10	10
	LD20	20
	LD60	60
	LD80	80
	LD90	90
	LD100	100
	LD150	150
	LD180	180
outer reef slope	WJ0	0
	WJ50	50

The grey background in the table was used to distinguish the data from Sansha Yongle Blue Hole and outer reef slope.

**Table 2:** Summary of sequences, operational taxonomic units (OTU) numbers, richness, and diversity index of eukaryote communities in different samples.

Samples	Raw Sequences	Effective Sequences	OTUs	Chao1	Shannon	Coverage (%)
LD0	82,584	80,265	1591	1573	7.62	99.7
LD10	96,121	93,102	1594	1567	6.07	99.5
LD20	95,414	93,308	1378	1351	3.94	99.6
LD60	81,004	76,761	41	39	1.64	100.0
LD80	90,493	85,745	1569	1550	4.73	99.5
LD90	94,964	77,212	1318	1311	8.14	99.8
LD100	87,143	82,482	3302	3312	9.14	99.5
LD150	82,515	68,851	1728	2591	7.55	99.5
LD180	91,558	85,053	1306	1242	3.21	99.6
WJ0	95,823	92,722	823	809	2.43	99.8
WJ50	84,171	82,270	1280	1278	5.54	99.8

**Table 3:** The relative abundance of eukaryotes in the top ten OTUs.

OTU ID	Species Information			LD0	LD10	LD20	LD60	LD80	LD90	LD100	LD150	LD180	WJ0	WJ50
	Phylum	Class	Order											
OTU1	Annelida	Polychaeta	Phascolosomatiformes	1.09	0.00	0.00	0.00	0.00	0.00	0.00	0.00	0.00	77.82	0.63
OTU2	Arthropoda	Arachnida	Araneae	0.00	0.00	0.00	75.28	0.00	0.00	0.00	0.00	0.00	0.00	0.00
OTU3	Entoprocta	Loxosomatidae *	Loxosomella **	0.14	0.23	0.08	0.00	0.22	0.08	0.11	0.09	60.12	0.00	0.00
OTU4	Alveolata	Dinophyceae	Gymnodiniales	0.30	4.13	44.01	0.00	0.53	0.29	0.36	0.85	0.18	0.00	0.08
OTU6	Ascomycota	Leotiomycetes	Thelebolales	7.21.	2.60	0.44	0.00	1.49	8.53	6.37	10.53	0.69	1.29	4.11
OTU5	Alveolata	Dinophyceae	Gymnodiniales	0.56	15.20	20.56	0.30	0.33	0.65	0.22	0.15	0.11	0.02	0.18
OTU7	Arthropoda	Hexanauplia	Cyclopoida	0.16	1.25	0.18	0.00	31.18	0.91	0.39	0.79	0.20	0.00	0.02
OTU8	Apicomplexa	Conoidasida	Gregarinasina	0.05	0.36	0.00	0.00	0.00	0.00	0.00	0.00	0.00	0.00	33.36
OTU9	Arthropoda	Unclassified	Unclassified	0.08	0.13	0.03	0.00	20.70	0.11	0.20	0.28	0.10	0.00	0.00
OTU10	Alveolata	Dinophyceae	Gymnodiniales	0.39	13.94	1.11	0.00	0.26	1.41	0.52	0.07	0.06	0.00	0.30

* at the family level. ** at the genus level.

**Table 4:** The richness and diversity of the eukaryotic community in the water column from the Sansha Yongle Blue Hole (YLBL) and the mid-region in the South China Sea (MRSCS).

Samples	Depth (m)	Chao1	Shannon	References
F3	25	3009.18	6.78	[49]
	75	2629.52	7.29	
	200	2956.26	7.59	
H7	5	2625.84	6.64	[49]
	25	3203.35	7.44	
	75	3170.70	7.43	
	200	3202.02	7.16	
YLBL	0	1762.85	7.41	this study
	10	1989.03	5.93	
	20	1662.53	3.74	
	60	28.00	1.29	
	80	1908.10	4.62	
	90	1489.51	7.96	
	100	3344.06	8.99	
	150	1698.22	7.29	
	180	1432.64	2.77	

The gray background in the table was used to distinguish the data from samples F3, H7 and YLBL.
